# Deciphering the Complexity: Addressing Recurrent Hamstring Injury in a Long-Distance Female Athlete

**DOI:** 10.7759/cureus.61961

**Published:** 2024-06-08

**Authors:** Tiago Simões-Moreira, Mafalda Mesquita-Guimarães, Mafalda Oliveira, Susana Moreira

**Affiliations:** 1 Physical Medicine and Rehabilitation, Unidade Local de Saúde de São João, Porto, PRT

**Keywords:** exercise, rehabilitation, sports medicine, hamstring injuries, athlete

## Abstract

Hamstring muscle injuries are prevalent in sports, presenting substantial hurdles for athletes and teams due to their high recurrence rates and prolonged recovery periods. The biceps femoris (BF) muscle is frequently implicated in these injuries, with notably high recurrence rates, particularly at the distal musculotendinous T-junction (DMTJ), where half of BF reinjuries occur. This paper presents a case study of a 31-year-old female long-distance athlete with a history of DMTJ rupture, shedding light on the intricacies associated with hamstring injuries. The case underscores the challenges in accurately interpreting imaging findings, reinforcing the need for clinical-imaging correlation and exploration of lesion mechanisms to achieve an accurate diagnosis, optimize outcomes, and facilitate a safe return to sport.

## Introduction

Hamstring muscle injuries are among the most common occurrences in sports, with an overall incidence ranging from 1.2 to four injuries per 1,000 hours of athlete exposure [1,2]. These injuries predominantly affect athletes engaged in football, rugby, soccer, track and field, and dance [3], resulting in significant time loss (averaging 24 days) and substantial costs for both professional athletes and teams. The biceps femoris (BF) muscle is primarily implicated in these injuries, with notably high recurrence rates, particularly at the distal musculotendinous T-junction (DMTJ), where half of BF reinjuries occur. Injury to the DMTJ of the BF is a distinct clinical entity that behaves differently from other hamstring injuries due to its complex, multicomponent anatomy and dual innervation [4]. There is no consensus on the mechanism of hamstring injury. Askling et al. [5] proposed two scenarios: injury during high-speed running and injury during stretching movements. High-speed running injuries typically affect the long head of the BF and have a shorter recovery time compared to stretching injuries, which commonly affect the semimembranosus muscle (SM) [6-8]. The running type of injury is the most frequent, and two theories on its mechanism exist in the literature.

One theory suggests hamstring vulnerability during active lengthening, often observed during the late swing phase of the running gait cycle [9]. Consequently, preventive studies have focused on eccentric strengthening, such as the Nordic hamstring exercises, which have been associated with significantly lower injury incidence. Runners typically require an average of 16 weeks to resume unrestricted sports activities following hamstring injuries [6]. Traditionally viewed as challenging for athletes due to prolonged rehabilitation periods and unpredictable return-to-sport timelines, hamstring muscle injuries warrant careful consideration. Recent research has highlighted differences in hamstring injury rates between male and female athletes [10,11]. Cross et al. found significant variations in hamstring strain injury rates between male and female intercollegiate soccer athletes, emphasizing the importance of understanding gender-specific risk factors [10]. Moreover, O'Sullivan et al. investigated hamstring injury rehabilitation and prevention strategies specifically tailored for female athletes, recognizing the need for gender-specific approaches in managing these injuries [11].

We present a case of a 31-year-old long-distance athlete with a history of previous DMTJ rupture, underscoring the complexities and challenges associated with hamstring injuries, as appearances can be misleading.

## Case presentation

**Figure 1:**
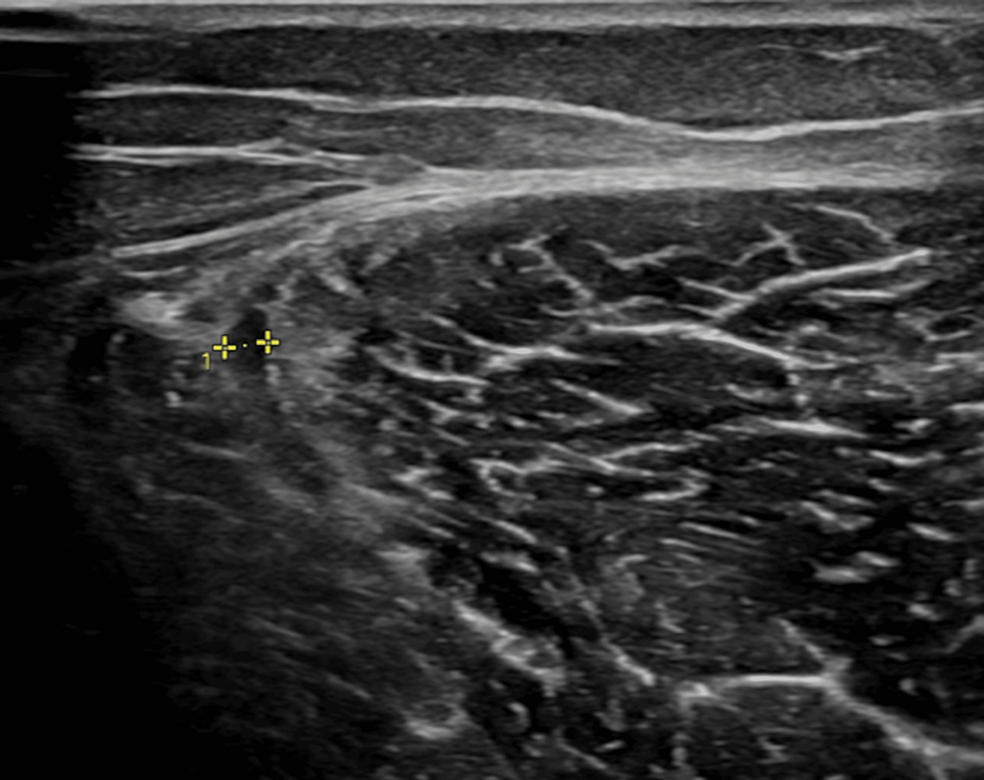
Musculoskeletal ultrasound of the left thigh: recurrent injury, with a rupture area of the peripheral aponeurosis of the distal junction of the long head of the biceps femoris (BF) (yellow *), associated with subaponeurotic edema and perifascial fluid lamina.

A 31-year-old female long-distance athlete presented with acute pain in the posterolateral region of her left thigh following a 400-m sprint as a part of her training for an upcoming marathon. On physical examination, tenderness was noted in the distal posterolateral thigh, along with discomfort during stretching and resisted knee flexion. Given her history of a previous rupture in the DMTJ of the BF with subsequent recurrence, further investigation was warranted. Musculoskeletal ultrasound was performed (Figure 1), revealing recurrent injury, with a rupture area of the peripheral aponeurosis of the DMTJ of the long head of BF (superficial zip), associated with subaponeurotic edema and a perifascial fluid lamina. The aponeurotic defect appeared to be small. As it seemed to be a new injury grafted onto scar tissue from previous injuries, she was advised to undergo a six-week rehabilitation program, including cross-training with an exercise bike, focusing on mobility and flexibility and gradually introducing strength training, starting with isometrics and progressing to concentric and eccentric isotonic training. In addition, it included low-level laser therapy (gallium-aluminum arsenide diode laser device with a wavelength of 800 nm, power output of 100 mv, continuous wave, applied at two points over the posterior left thigh, for three minutes each, producing energy of approximately 40 J/cm^2^) and therapeutic ultrasound (pulsed mode, frequency of 1 MHz, intensity of 1 W/cm^2^, for five minutes), both applied twice a week for a total of 12 sessions. During the gradual return to running, the athlete experienced continued discomfort and pain in the distal posterolateral thigh. She repeated the ultrasound, whose images were similar to the ones at the beginning of the complaints. These findings prompted her to undergo magnetic resonance imaging (MRI) for further characterization of the injury. The MRI report identified sequelae of the previous BF rupture, as well as an additional rupture in the semitendinosus (ST) muscle of 19 mm at the mid-thigh.

**Figure 2:**
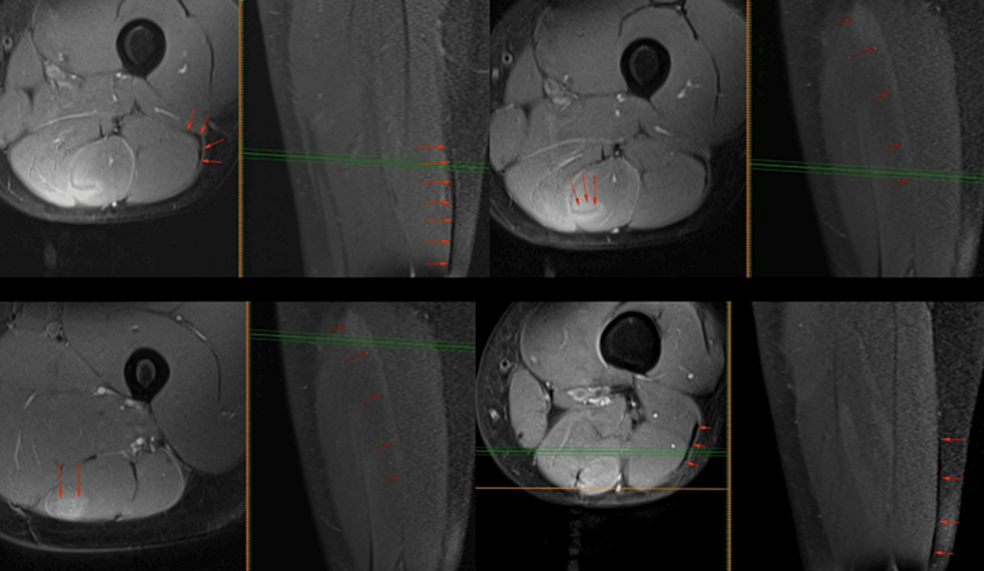
T2-weighted MRI imaging of the left thigh: transverse and coronal planes - para-aponeurotic edema within the semitendinosus muscle, particularly concentrated in the intramuscular raphe and aponeurosis of the distal musculotendinous T-junction (DMTJ) (red arrows).

Given that the pain and discomfort were in a more distal region of the thigh, and the athlete remained without introducing speed variations or any associated load into her training, this situation raised doubts about a possible clinical-imaging incongruence. A reevaluation of the MRI images (Figure 2) was requested by a specialist in musculoskeletal radiology, who related complete scar tissue maturation and remodeling in the BF, alongside with thickening of the peripheral muscular aponeurosis at the DMTJ. Para-aponeurotic edema within the ST muscle, particularly concentrated in the intramuscular raphe and aponeurosis of the DMTJ, consistent with exercise-induced edema, was described. Importantly, no evidence of muscle fiber or aponeurosis rupture, nor structural damage associated with the edema, was detected. The athlete continued her rehabilitation program for an additional two weeks. Four weeks after the diagnosis, she fully recovered and completed a 10 km race without experiencing any pain or discomfort. She maintains daily exercises with a physical trainer to enhance her flexibility, muscle strength, and running technique. In addition, she has annual follow-up appointments with a sports medicine specialist.

## Discussion

Hamstring injuries are widespread across various sports, imposing significant challenges on athletes and teams due to the loss of time and financial burdens they entail. It is imperative to comprehend the frequency of these injuries to effectively address them. A key aspect of hamstring injuries is the involvement of the BF muscle, particularly at the DMTJ, where recurrence rates are notably high. The mechanisms behind hamstring injuries remain contentious, with proposed scenarios including high-speed running and stretching movements. Stressing the importance of biomechanical understanding is crucial for devising preventive measures, such as eccentric strengthening exercises, like the Nordic hamstring exercises. The present case report further enriches the discussion by illustrating the intricate challenges encountered in the diagnosis, management, and follow-up of hamstring injuries, particularly when addressing an injury with specific characteristics such as DMTJ of the BF. Despite initial interventions, ongoing discomfort and discrepancies in clinical imaging urged a revision of the MRI images by a specialized musculoskeletal radiologist. This reevaluation revealed that what appeared to be an SM lesion was a para-aponeurotic edema within the semitendinosus muscle, particularly concentrated in the intramuscular raphe and aponeurosis of the distal MTJ, consistent with exercise-induced edema, allowing the athlete to gradually resume her training activity.

## Conclusions

The case underscores the complexities associated with hamstring injuries, as appearances can be deceptive, highlighting the challenges in accurately interpreting imaging findings, reinforcing the need for clinical-imaging correlation and exploration of lesion mechanisms to achieve an accurate diagnosis, such as the importance of a multidisciplinary approach to recurrent injuries in athletes. This will contribute to a comprehensive assessment and tailored management strategies. By understanding the underlying mechanisms of the injury, clinicians can optimize treatment approaches, mitigate risks of recurrence, and facilitate a safe return to sport for athletes.
